# The male sling for stress urinary incontinence: tips and tricks for success

**DOI:** 10.1590/S1677-5538.IBJU.2020.1122

**Published:** 2021-02-28

**Authors:** Brian M. Inouye, Hayley A. Premo, Dane Weil, Andrew C. Peterson

**Affiliations:** 1 Duke University Medical Center Division of Urologic Surgery DurhamNC USA Division of Urologic Surgery, Duke University Medical Center, Durham, NC, USA; 2 Charles George VA Medical Center Division of Urologic Surgery AshevilleNC USA Division of Urologic Surgery, Charles George VA Medical Center, Asheville, NC, USA

## Abstract

Urethral slings are a good treatment option for mild male stress urinary incontinence. There are many different sling options, but herein our group describes our techniques with the Advance^®^ and Virtue^®^ slings. More important than technique, we strongly think that patient selection is paramount to sling success. We only offer slings to patients who have low 24 hour pad weights, high Valsalva leak point pressure, and no history of pelvic radiation. Still, like with any surgery, we recommend that the surgeons implant the device that they are most comfortable with along with their chosen techniques.

## INTRODUCTION

Urinary incontinence has a considerable impact on the quality of life for men. It is estimated to affect 4.8% of males ages 19-44 and up to 32.3% of men over the age of 65. Of these patients, an average of 2.67% are impacted by stress urinary incontinence (SUI) (1). SUI is the involuntary leakage of urine with transient increases in intraabdominal pressure, such as with sneezing, coughing, or lifting (2). SUI is a common health issue that has a considerable impact on patient quality of life for men. Radical prostatectomy is currently the most common cause of male SUI (3). The etiology of PPI is thought to be multifactorial, arising from a combination of detrusor hypocontractility, intrinsic sphincter dysfunction, and decreased membranous urethral length (3). Because so many men are affected by SUI, there are many different treatment options available.

### Non-sling treatments for stress urinary incontinence

There are many non-invasive and invasive treatment options for male SUI. Non-invasive options include behavioral therapy (lifestyle change, timed voiding, etc.), pelvic physical therapy, penile clamps, and condom catheters among other urinary collection methods (4). However, many men eventually choose to pursue surgical correction. While the artificial urinary sphincter remains the gold standard for SUI, the male sling has emerged as another treatment for a specific group of men with SUI.

### The male sling for stress urinary incontinence

While the original male slings depended heavily on retropubic placement and bone anchored systems, contemporary devices vary through approach and whether the mesh is fixed or adjustable (Table-1) (5).

**Table 1:** Commercially Available Male Urethral Slings across the World.

Name of Sling (Company, Location)	Approach	Adjustable Mesh
Advance XP^®^ (Boston Scientific; Minnetonka, MN, USA)	Transobturator	Fixed
Virtue^®^ (Coloplast, Humlebaek, Denmark)	Transobturator and prepubic	Fixed
I-STOP TOMS^®^ (CL Medical, Lyon, France)	Transobturator	Fixed
Argus^®^ (Promedon, Cordoba, Argentina)	Retropubic	Adjustable
Argus-T^®^ (Promedon, Cordoba, Argentina)	Transobturator	Adjustable
Male ReMeex^®^ (Neomedic, Barcelona, Spain)	Retropubic	Adjustable
ATOMS^®^ (A.M.I. Surgical, Vienna, Austria)	Transobturator	Adjustable

Our group is most familiar with the AdVance^®^, newer Advance XP^®^, and Virtue^®^ slings. The AdVance XP^®^ (Boston Scientific, Minnetonka, MN) is a transobturator sling (6). In a study by Grabber et al., the cure rate for the AdVance XP^®^ was 66.7% which is comparable to that of the original AdVance^®^ sling (6). In an international prospective study of the Advance^®^ sling, the authors defined success as the patient having no urinary leakage while improvement included a reduction in leakage and pad usage with a maximum of 1 pad per day (7). At 24 months, 90% of participants self-reported improvement in their condition. Objectively, 80.6% used one or no pads daily. While the treatment failure rate was 19.4%, no complications arose that necessitated sling removal.

The Virtue^®^ Sling (Colo, plast Humlebaek, Denmark) is a quadratic transobturator sling that theoretically provides bidirectional support and compression of the bulbar urethra (8). In a study by Ferro et al., the efficacy of the Virtue^®^ sling was tested in patients that had mild-moderate SUI. At 12 months follow-up, the majority of patients (82.7%) were using no pads and the rest only utilized 1 per day. Unfortunately, the patient's dryness was not durable as dryness decreased to 58.6% at 36 months. Still, all patients reported continued satisfaction with the improvement in their PPI (8).

### Mechanism of male sling

Our group feels that understanding the mechanism of action by the male sling is paramount to understanding patient selection, optimal placement techniques, and thus better outcomes. Available mechanistic data on the transobturator sling suggests that it provides an anatomical change to the urethra by lengthening it (9). Rehder and Gozzi examined the transobturator sling in cadavers and found that urethral bulb was elevated proximally lengthening the functional membranous urethra from a mean of 3mm to 17.2mm (10). More recently, Kahokehr et al. used MRI measurements to show that the sling increases the functional urethral length by increasing the distance between the vesicourethral anastomosis and the bulbar urethra to greater than 15mm (9).

### The importance of patient factors

By understanding that slings require pliable anatomy to succeed, our group has found that more than the techniques in sling placement, the most important factor leading to a good outcome is correct patient selection. The most basic tenant is to only offer slings to men who have low 24 hour pad weights, however, “low” is a subjective term. Our group has found similar results to Fischer et al., that 24 hour pad weight of less than 400cc was associated with higher rates of success (11).

We also reserve slings for men that have a Valsalva leak point pressure (VLPP) greater than 60cmH2O. Our group has previously demonstrated that VLPP greater than 70cmH2O is an indicator of success, but anecdotally we have found that patients with greater than 60cm water pressure also do well (12). Furthermore, Barnard et al. found that a VLPP greater than 100cmH2O, compared to less than 100cmH2O, was associated with a higher degree of success (13).

Finally, we do not offer a sling to men who have a history of pelvic radiation. While success has been reported to be 51-73% with the AdVance^®^ sling, Bauer et al. found a cure rate of just 25% and a failure rate of 50% in irradiated patients (14, 15). Our group thinks that there are enough studies to suggest poor sling outcomes in men with a history of radiation. While most studies have been underpowered, both Rehder et al. and Cornu et al. found a higher failure rate in irradiated patients compared to non-radiated (16, 17).

### Preoperative workup

Acknowledging these positive predictive patient factors, we supplement the post-prostatectomy incontinence AUA guidelines workup for patients who desire surgical treatment for SUI. The most important part of the preoperative workup is the history and physical. One must know the type of incontinence, radiation status, daily pad usage with an estimate of volume of loss per day, that bother to the patient. Physical exam is needed to demonstrate the presence and severity of SUI.

We then perform non-invasive and invasive tests to help elucidate the type and severity of incontinence. Non-invasive testing includes bladder diary, pad weight, and urine culture. Invasive testing includes cystoscopy to rule out bladder pathology and characterize the sphincter at rest and urodynamics to understand the VLPP and if there is any detrusor overactivity that may benefit from medical management.

### AdVance XP^®^ sling technique

Positioning and Prep: We position the patient in dorsal lithotomy and perform a ten-minute chlorhexidine scrub followed by a chlorhexidine and alcohol-based paint. In preparing the sling itself, we do not let it sit in saline prior to implantation as we have found that the white cardboard can get flimsy and be difficult to remove when removing the lateral sheaths. Patients receive perioperative antibiotics tailored to preoperative urine culture, local antibiogram, and accordance with American Urological Association guidelines.

Urethral Dissection: When we perform the perineal urethral dissection, we find it necessary to take down some of the central tendon to allow the bulbar urethra freedom to move cranially and thus lengthen the functional urethral length. The most proximal portion of the central tendon is left in place to serve as a crux for the sling to pull on when advanced. We feel that this prevents the sling from slipping behind the bulb of the urethra and therefore resulting in delayed failures.

Sling Placement and Tensioning: Prior to placement of the sling trocar, we infiltrate the transobturator space with lidocaine with epinephrine through a spinal needle. This not only helps in identifying the obturator fossa but also hydrodissects, decreases postop pain, and decreases troublesome bleeding with the addition of epinephrine. Next, we pass the trocar by placing it at a 45° angle toward the incision, toeing in to get through obturator, dropping the angle to 10°, and rotating it out onto our finger to bring the needle out as anterior as possible without damaging the urethra. After placement of the sling per the manufacturer's instructions we do perform cystoscopy while we tension it so to not coapt the urethra too much and cause postoperative urinary retention. We have not found utility in placing anchoring stitches.

Intraop Complications: If trocar passage leads to hemorrhage, we compress simultaneously on the stab/trocar incision as well as laterally from inside the perineal wound. If bleeding from the trocar site does not cease, we leave the sling sheaths intact through the lateral stab incisions and inject a gelatin-thrombin matrix into the sheath at the end of the case for hemostasis. If there is central tendon bleeding, holding pressure for five minutes typically stops any bleeding.

Postoperative Management: We leave a Foley catheter overnight to allow swelling to decrease. Patients do not receive postoperative antibiotics. They are told to not lift more than ten pounds and to minimize sitting for prolonged periods of time for six weeks. Five to 7% of men may experience transient acute urinary retention after placement of the sling. We customarily manage this with replacement of a Foley catheter for another 24 to 72 hours. If spontaneous voiding is not obtained at that time we then teach the patient intermittent catheterization. All cases of retention in our hands have resolved spontaneously within 2 to 4 weeks and we have yet to perform sling lysis for postoperative retention.

### Virtue^®^ sling technique

Positioning and Prep: We position the patient in dorsal lithotomy and perform a ten-minute chlorhexidine scrub followed by a chlorhexidine and alcohol-based paint. Patients receive perioperative antibiotics tailored to preoperative urine culture, local antibiogram, and accordance with American Urological Association guidelines.

Urethral dissection: The bulbo-spongiosus muscle is left intact on the urethra during the perineal dissection, however, we find it necessary to take down some of the central tendon to allow the bulbar urethra freedom to move cranially and thus lengthen the functional urethral length. The very most proximal portion of the central tendon is left in place to serve as a crux for the sling to pull on when advanced. We feel that this prevents the sling from slipping behind the bulb of the urethra and therefore resulting in delayed failures.

Sling Preparation: To help with axial compression we place four transverse rows of zero prolene corset stitches through the sling.

Sling Placement and Tensioning: We pass the transobturator arm J-hook trocars from inside the perineal wound, around the inferior pubic ramus, and to the skin. We then place a deep central tacking stitch to anchor the sling to the central tendon to prevent distal migration. Prior to passing the prepubic arms, we pre-place a tacking stitch through the periosteal tissue and where we estimate the prepubic arm to lie once on tension. We then pass the J-hook outside to in to grasp the prepubic arms. Once they are pulled through, we hold tension and tie down our pre-placed periosteal stitches. We then put the transobturator arms on tension and place inferior ramus periosteal stitches that anchor these arms. Next, we place three more tacking stitches through the mesh and spongiosum between the corset stitches to anchor the mesh to the urethra. Finally, under cystoscopic vision, we tie down the original four rows of mesh corset stitches to compress the urethra.

Intraop Complications: Similar to management of intraop bleeding during AdVance XP^®^ placement, if there is bleeding with trocar passage we first compress and if this does not stop we place Floseal^®^. If there is central tendon bleeding, holding pressure for five minutes typically stops any bleeding.

Postoperative Management: We leave a Foley catheter overnight to allow swelling to decrease. Patients do not receive postoperative antibiotics. They are told to not lift more than ten pounds and to minimize sitting for prolonged periods of time for six weeks.

## CONCLUSION

Male urethral slings are a great surgical option for the patient with mild stress urinary incontinence without a history of radiation therapy. There exist many different types of slings and techniques, and our tips are presented here. We feel one of the most important components of success with this type of procedure is appropriate patient selection as outlined above. Still, like with any surgery, we recommend that the surgeon implant the device that they are most comfortable with along with their chosen techniques.
